# Supplementary material for: Antifouling graphene oxide membranes for oil-water separation via hydrophobic chain engineering
Source: Nat Commun. 2022 Nov 28;13:7334. doi: 10.1038/s41467-022-35105-8 (PMC9705527; doi:10.1038/s41467-022-35105-8)
Supplement: Supplementary file 1 — Supplementary Information [file 41467_2022_35105_MOESM1_ESM.pdf]

Supplementary Information for

Antifouling graphene oxide membranes for oil-water separation via hydrophobic chain engineering

Chao Yang[1], Mengying Long[1], Cuiting Ding[1], Runnan Zhang[1, 2, 3], Shiyu Zhang[4], Jinqiu Yuan[1], Keda Zhi[1], Zhuoyu Yin[1], Yu Zheng[1], Yawei Liu[5, *], Hong Wu[1, 2, 3, 6, *], Zhongyi Jiang[1, 2, 3, 4, *]

[1] Key Laboratory for Green Chemical Technology, School of Chemical Engineering and Technology, Tianjin University, Tianjin 300072, China

[2] Zhejiang Institute of Tianjin University, Ningbo, Zhejiang 315201, China

[3] Haihe Laboratory of Sustainable Chemical Transformations, Tianjin 300192, China

[4] Joint School of National University of Singapore and Tianjin University, International Campus of Tianjin University, Binhai New City, Fuzhou 350207, China

[5] Beijing Key Laboratory of Ionic Liquids Clean Process, CAS Key Laboratory of Green Process and Engineering, State Key Laboratory of Multiphase Complex Systems Institute of Process Engineering, Chinese Academy of Sciences, Beijing, 100190, China

[6] Tianjin Key Laboratory of Membrane Science and Desalination Technology, Tianjin University, Tianjin 300072, China

* Corresponding authors.

E-mail addresses: ywliu@ipe.ac.cn (Yawei Liu), wuhong@tju.edu.cn (Hong Wu), zhyjiang@tju.edu.cn (Zhongyi Jiang).

Inventory of Supporting Information:

Supplementary Methods

Supplementary Figures 1-14

Supplementary Tables 1-3

Supplementary Reference 1-24

Supplementary Methods

Characterization

Characterization of chemical structures and morphology

Surface morphology and topography were observed via a scanning electron microscope (SEM, Apreo S LoVac, FEI) and an atomic force microscope (AFM, XE-70, Park). The element distribution of the membranes was carried out by transmission electron microscopy (TEM, Tecnai G2 F20, FEI) which is equipped with an energy dispersive spectrometer (EDS, GATAN EELS GIF Tridiem 863). The chemical structures were detected by Fourier transform infrared spectroscopy (FTIR, Nicolet 560) and X-ray photoelectron spectroscopy (XPS, K-Alpha+).

Contact angles

Contact angles (CA), including in-air water CAs (WCA) and underwater oil CAs (OCA), were obtained by a contact angle goniometer (OCA15EC, Dataphysics). A drop of water or oil (1 μL) was placed on the membrane surfaces using a micropipette. When equilibrium was reached, the values were obtained by measuring the angle between the liquid droplets and the membrane surfaces.

Surface energy

The surface energy was calculated according to the two-liquid Owens–Wendt–Kaelble model [1]. The CAs of water and diiodomethane were tested, respectively. The total (γ), polar (γ^{p}), and dispersive (γ^{d}) energies were obtained as the following equations:

$$\gamma = \gamma^{p} + \gamma^{d} \qquad (1)$$

$$\gamma(1 + cos\theta) = 2\sqrt{\gamma^{P}\gamma_L^{P}} + 2\sqrt{\gamma^{d}\gamma_L^{d}} \qquad (2)$$

where θ, γ, and γ_L is the liquid CA on the membrane and the surface energy of membrane surfaces and liquids, respectively.

Underwater oil adhesion force

AFM is used to test the adhesion of oils on the membrane surfaces. Hexadecane, commonly used target oil pollutants, was modified on the detector tip of AFM. The hexadecane-modified detector tip was touched the membrane surfaces and then retracted from the membrane surfaces underwater, during which the force of the cantilever is measured, and the maximum force is defined as the adhesion force.

Differential scanning calorimetry measurement

Differential scanning calorimetry (NETZSCH DSC 200 F3) was used to monitor the crystallization temperature and crystallization enthalpy of water on the membrane surfaces. The samples were prepared by putting ~10 mg of water onto the membrane surfaces with a diameter of 6 mm and making the water fully wet the entire membrane surfaces. Then, the samples were tightly sealed in aluminum pans. The sample cells were cooled from 20 °C to -40 °C at a rate of 5 °C/min and then heated to 20 °C at the same rate.

Separation and antifouling performances evaluation

The separation and antifouling performances of the membranes were evaluated using a dead-end filtration facility. All tests were performed under 25 °C and 2000 rpm magnetic stirring. The membranes were first pre-compacted at 1 bar for 30 min, and the flux and permeance were carried out at 0.5 bar and calculated via Eq. 3 and Eq. 4 [2, 3].

$$Flux = \frac{V}{A \times t} \qquad (3)$$

$$Permeance = \frac{V}{A \times t \times P} \qquad (4)$$

where V, A, t, and P are the volume of filtrate (L), the effective membrane area (m^2), the running time (h), and the transmembrane pressure (bar), respectively.

The rejection of the membranes was tested by filtering emulsions. The emulsions, typically, were prepared by mixing 1 mL hexadecane and 99 mL water under 700 rpm mechanical stirring for 12 h.

The size distribution of the emulsions was gained via a dynamic light scattering (Zetasizer nano ZS90, UK). The rejection is calculated by Eq. 5.

$$Rejection = \left(1 - \frac{c_f}{c_0}\right) \times 100\% \qquad (5)$$

where c_f and c_0 are the concentration of the filtrate and the feed emulsions. The oil concentration was obtained by detecting the total organic carbon content with a total organic carbon analyzer (OI, 1030W+1088, O·I·Anealytical).

The antifouling performances of the membranes were tested by filtering emulsions at 0.5 bar. Typically, the membranes were pre-compacted with water at 1 bar for 30 min. Then, pure water was filtered through the membrane for 30 min and the average water flux of the last 10 min was recorded as J_{w1}. Then, emulsions were filtered through the membrane for 30 min. The average flux of the last 10 min was recorded as J_p. After washing with pure water for 30 min, water fluxes of the membranes were tested and recorded as J_{w2}. The flux recovery ratio (*FRR*) and total flux decline ratio (DR_t) of the membranes were calculated by Eq. 6 and Eq. 7.

$$FRR = \frac{J_{w2}}{J_{w1}} \times 100\% \qquad (6)$$

$$DR_t = \left(1 - \frac{J_p}{J_{w1}}\right) \times 100\% \qquad (7)$$

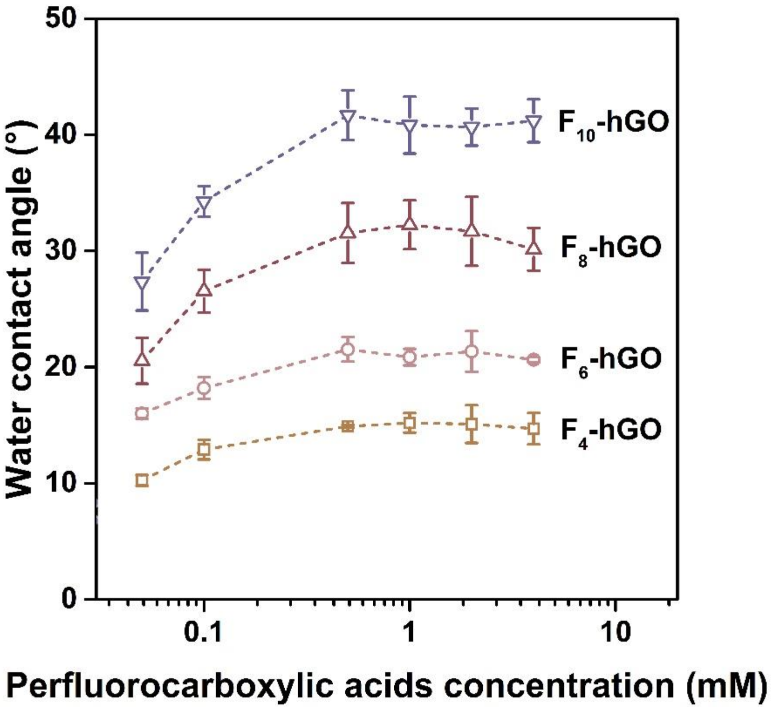

Supplementary Figure 1. Water contact angle of the F-hGO membrane varied with the concentration of perfluorocarboxylic acids solution. All error bars in this figure represent standard deviations for 3 measurements. Source data are provided as a Source Data file.

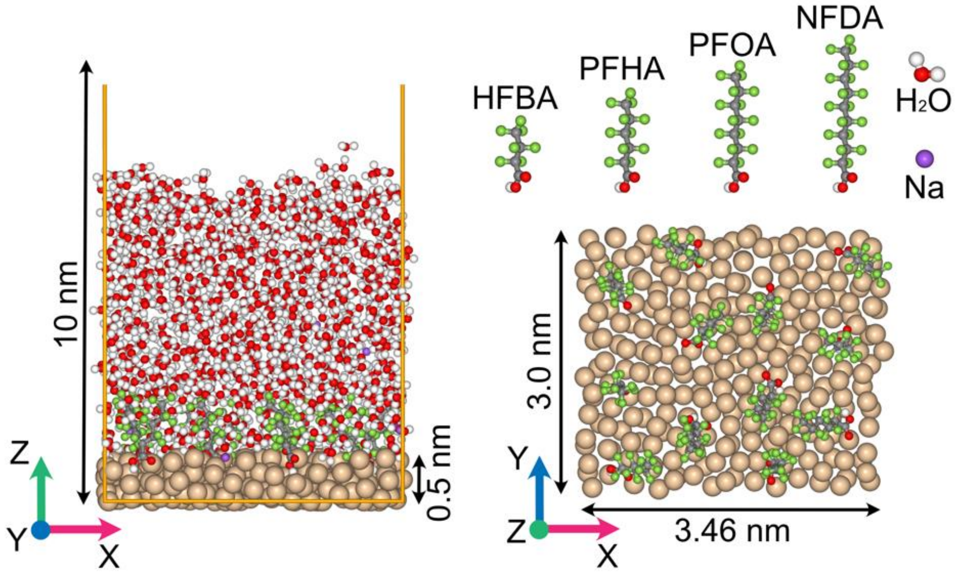

Supplementary Figure 2. A typical snapshot of the box used in our simulations (its edges $L_x =$ 3.46 nm, $L_y = 3.0$ nm and $L_z = 10$ nm). Water molecules and ions are on the modeled substrate with perfluoroalkyl chains. The top view of the substrate with perfluoroalkyl chains and molecular structures of each species in the box are also given.

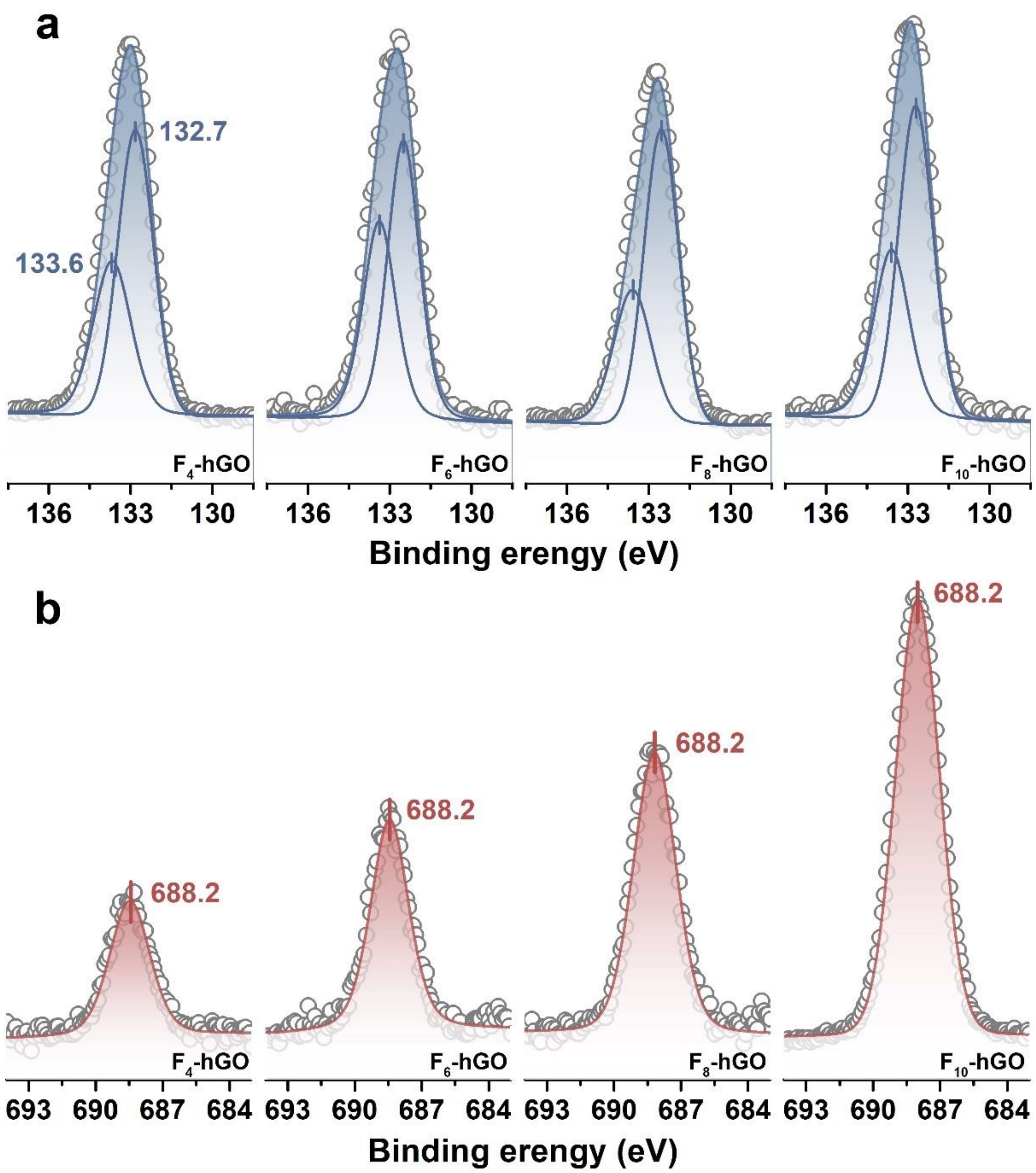

Supplementary Figure 3. (a) P *2p* high-resolution XPS spectra of the hGO and F-hGO membranes. **(b)** F *1s* high-resolution XPS spectra of the F-hGO membranes. Source data are provided as a Source Data file.

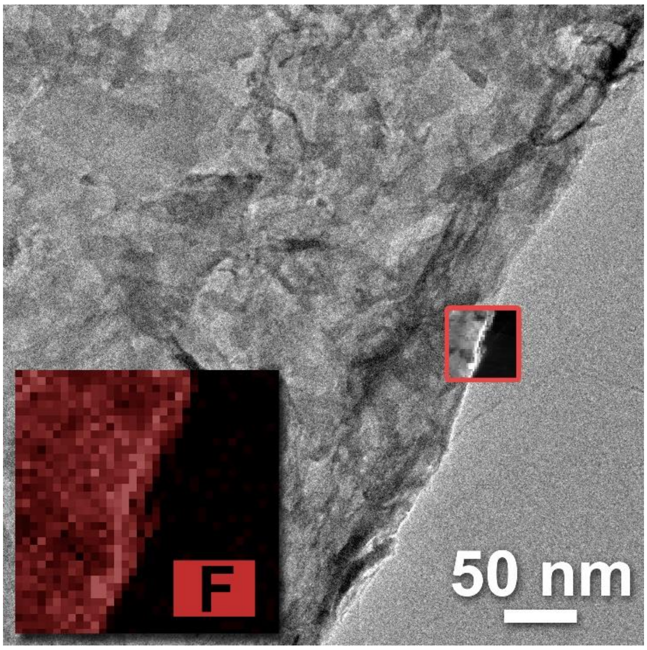

Supplementary Figure 4. Transmission electron microscopy and corresponding energy dispersive X-ray spectroscopy mapping images of the F_6-hGO membrane without the substrate.

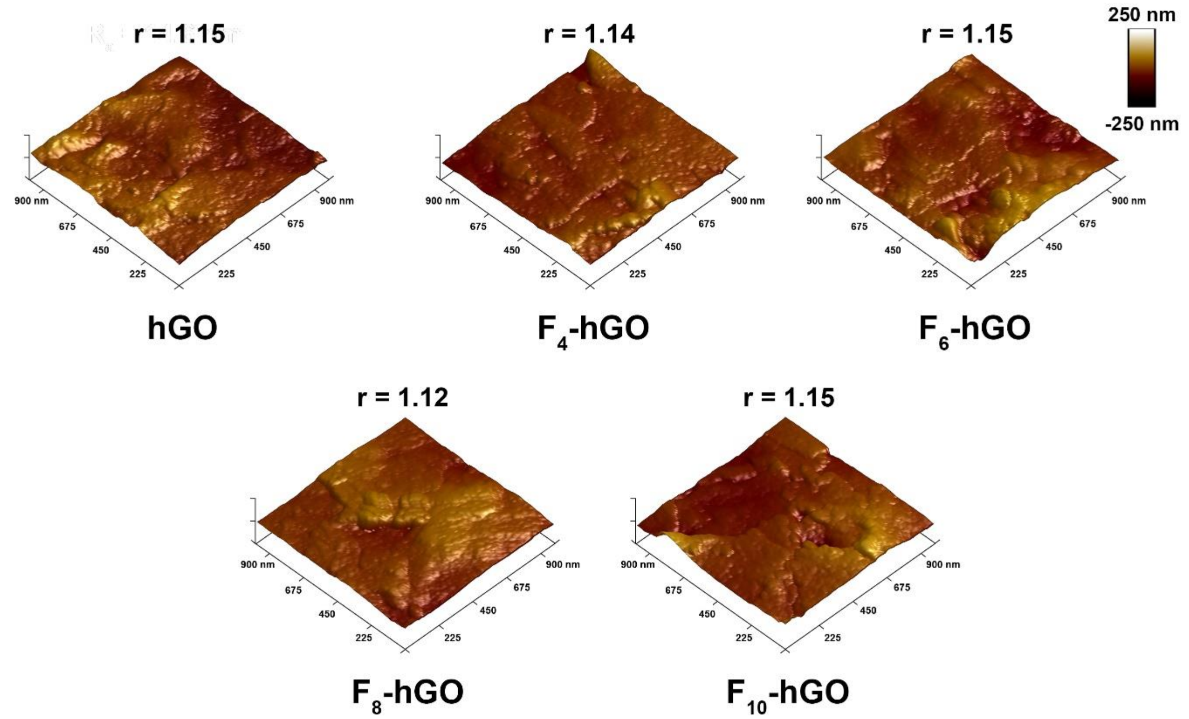

Supplementary Figure 5. AFM topography and roughness factor (r) of the surfaces of the hGO and F-hGO membranes.

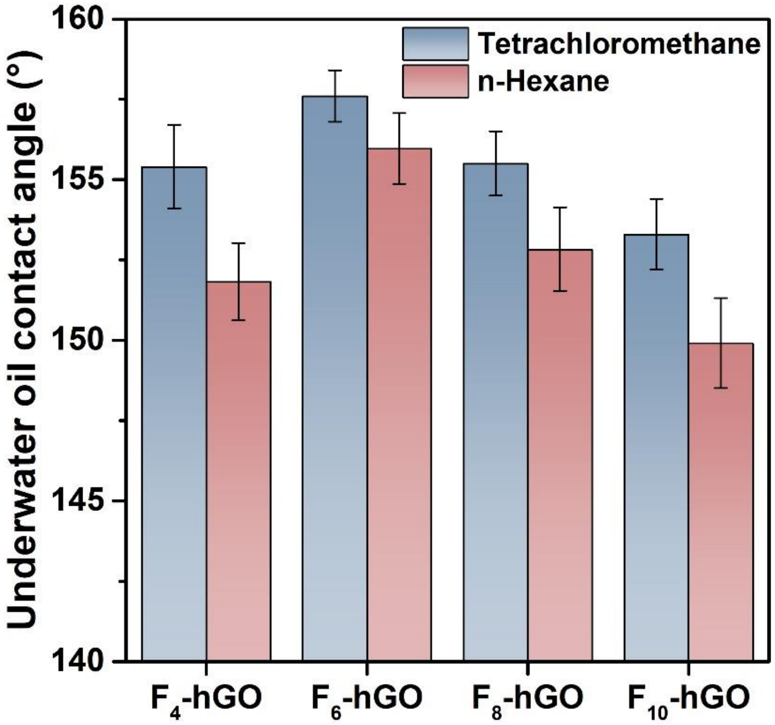

Supplementary Figure 6. Underwater oil contact angles of the F-hGO membranes. The probe oils are tetrachloromethane and n-hexane. All error bars in this figure represent standard deviations for 3 measurements. Source data are provided as a Source Data file.

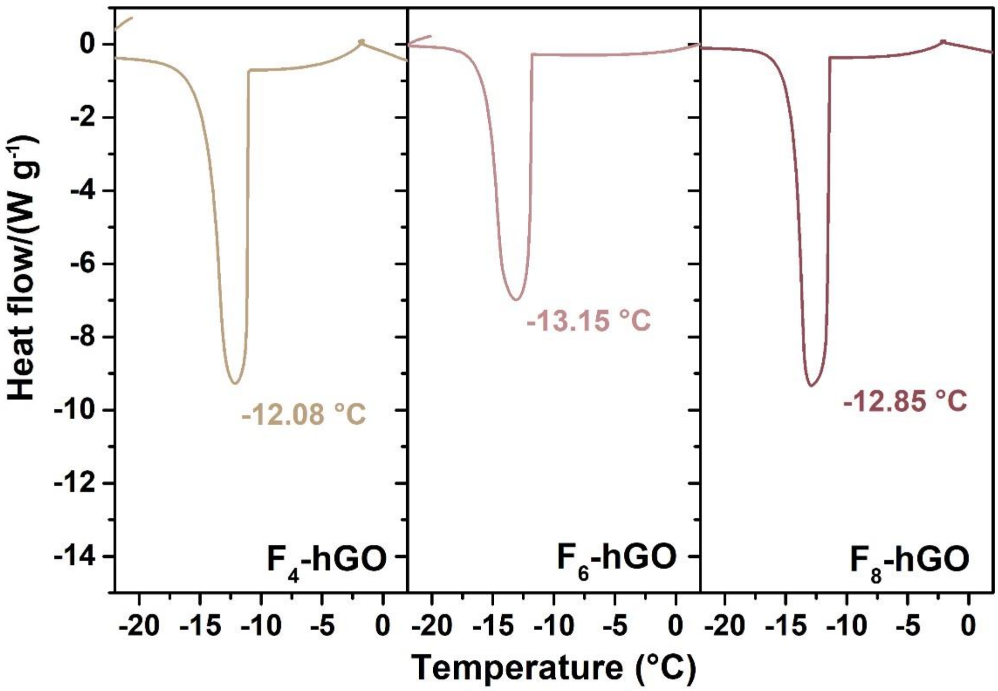

Supplementary Figure 7. Differential scanning calorimetry curves of water (~10 mg) on the F_4-hGO, F_6-hGO, and F_8-hGO membranes. Source data are provided as a Source Data file.

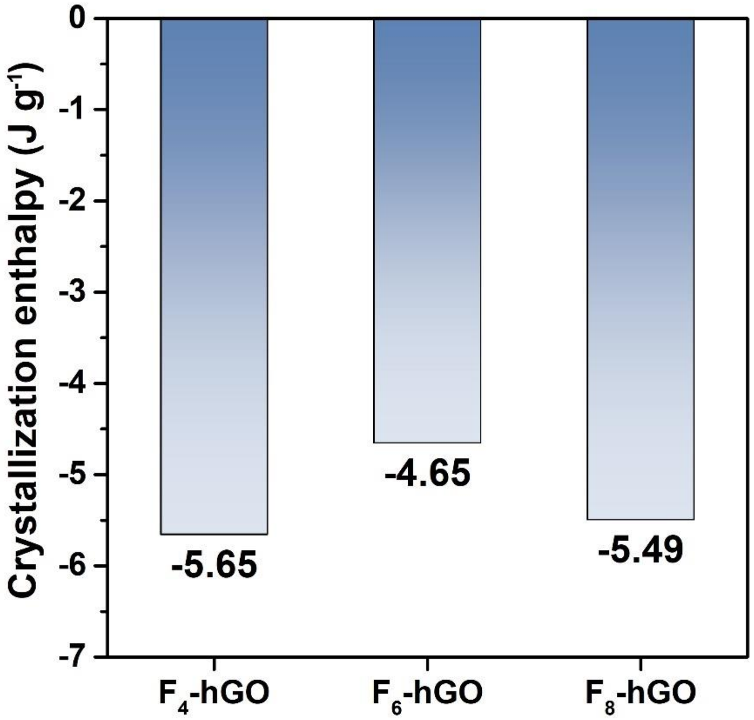

Supplementary Figure 8. Crystallization enthalpy of water (~10 mg) on the F_4-hGO, F_6-hGO, and F_8-hGO membrane. Source data are provided as a Source Data file.

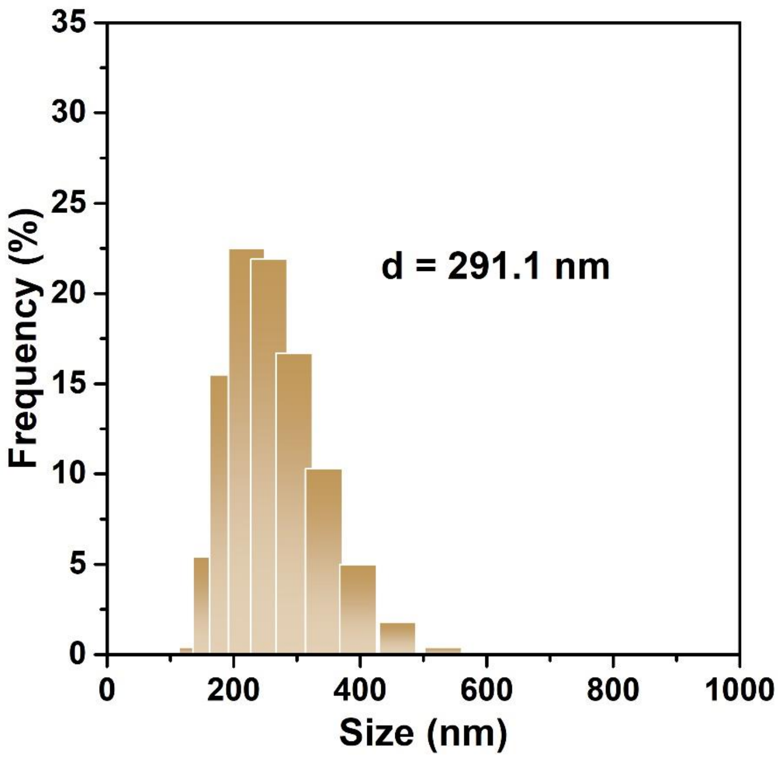

Supplementary Figure 9. Size distribution of the hexadecane-in-water emulsion (1000 ppm).

Source data are provided as a Source Data file.

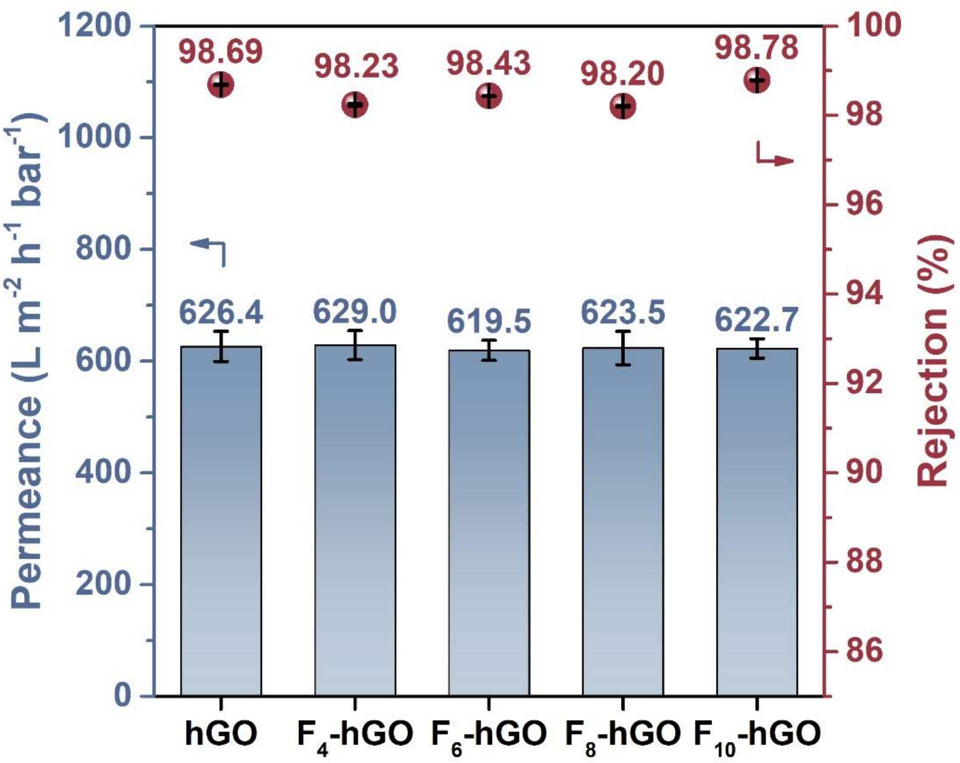

Supplementary Figure 10. Separation performances of the hGO and F-hGO membranes. The targeted oily wastewater is hexadecane-in-water emulsion (1000 ppm). All error bars in this figure represent standard deviations for 3 measurements. Source data are provided as a Source Data file.

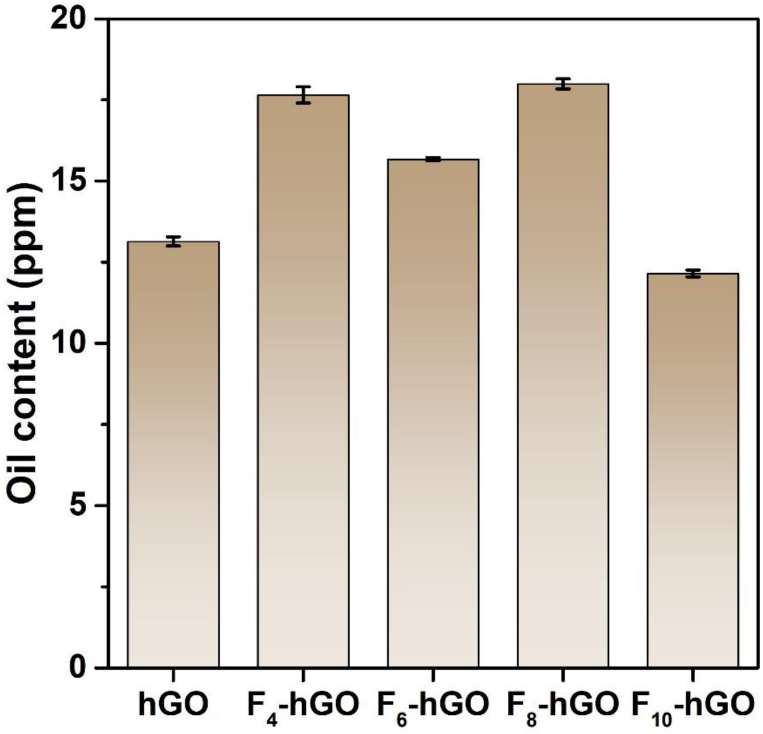

Supplementary Figure 11. Oil contents of the filtrate after treating by the hGO and F-hGO membranes. All error bars in this figure represent standard deviations for 3 measurements. Source data are provided as a Source Data file.

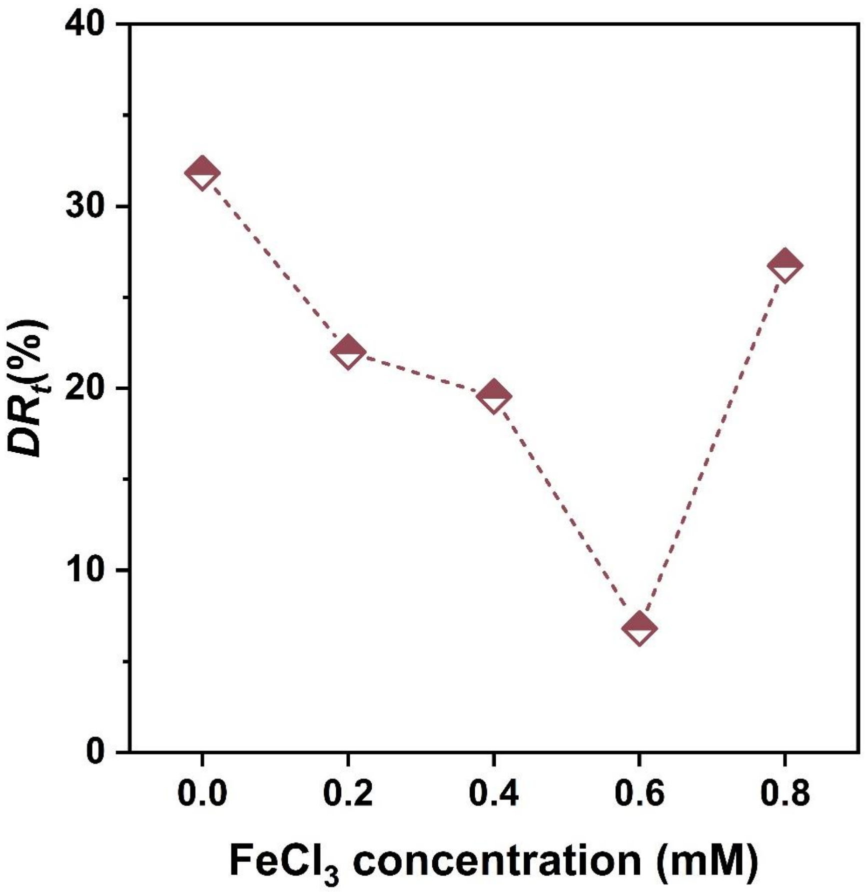

Supplementary Figure 12. DR_t of the F_6-hGO membrane prepared at the different concentration of the immersed $FeCl_3$ solution. The targeted oily wastewater is hexadecane-in-water emulsion (1000 ppm). Source data are provided as a Source Data file.

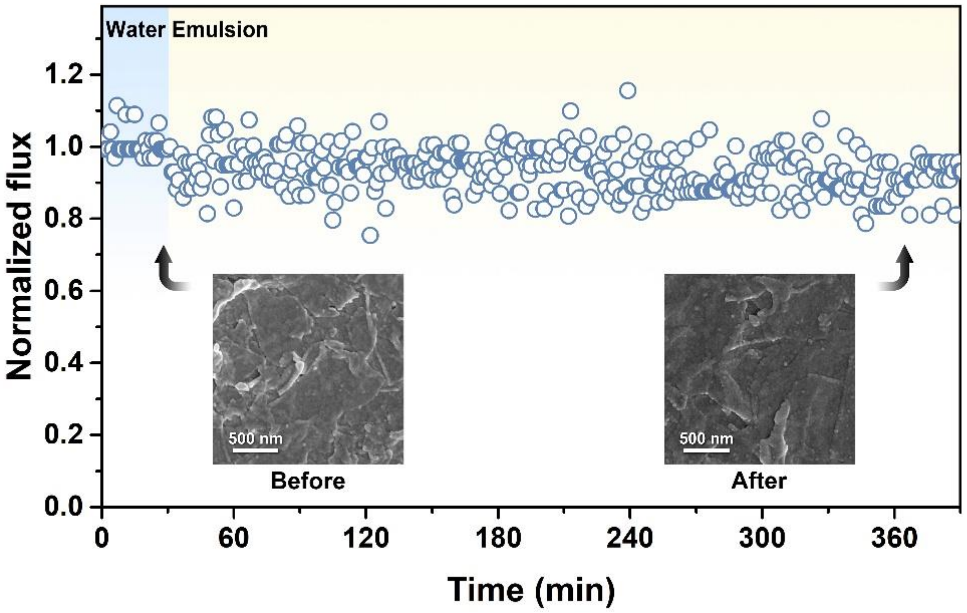

Supplementary Figure 13. Time-dependent normalized flux of the F_6-hGO membranes during scaling experiment. The insert is the surface morphology of the F_6-hGO membrane before and after the scaling experiment. Source data are provided as a Source Data file.

The gypsum scaling experiment was performed. The hexadecane-in-water emulsion with 20 mM sodium sulfate and 20 mM calcium chloride was used as the feed solution to conduct the filtration. After treating the emulsion for 6 h, no significant flux decline was observed as presented in Figure S12. The surface morphology of the membrane before and after the scaling experiment was tested by SEM. As shown in the insert of Figure S12, no gypsum scaling can be found on the membrane surface.

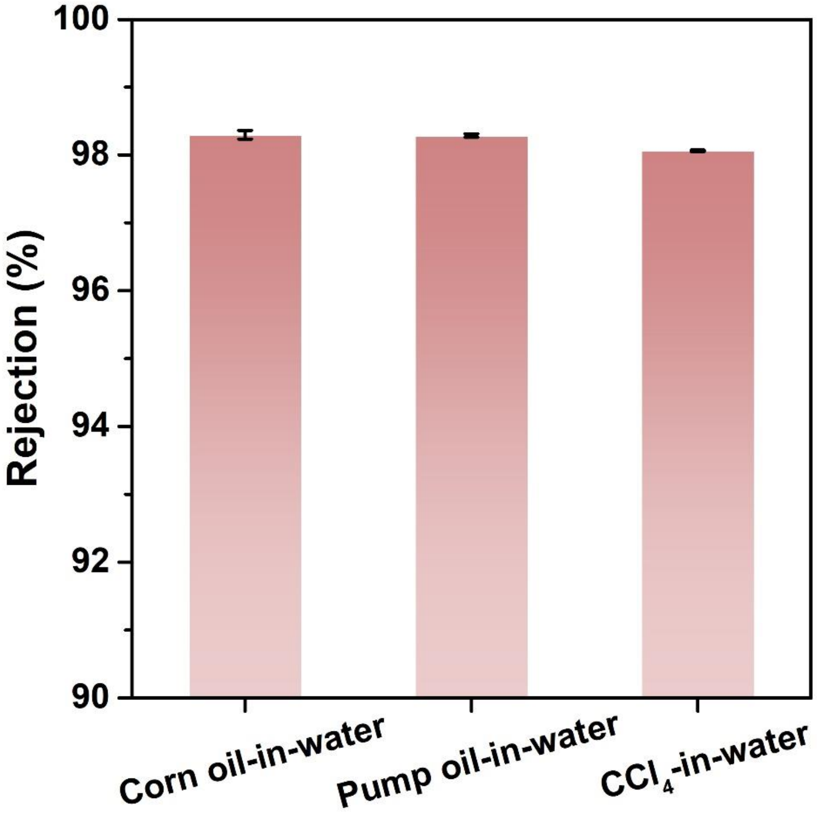

Supplementary Figure 14. Rejection of the F_6-hGO membrane for treating different oil-in-water emulsion (1000 ppm). All error bars in this figure represent standard deviations for 3 measurements. Source data are provided as a Source Data file.

Supplementary Table 1. Experimental and calculated underwater oil contact angles of the hGO, and F-hGO membranes

Membrane	γ_{SW} (mN m^{-1})	γ_{SO} (mN m^{-1})	γ_{OW} (mN m^{-1})	θ'^{cal}_{OW} (°)	θ'^{exp}_{OW} (°)
hGO	9.8	17.4	51.0	164.7	168.4
F_4-hGO	0.8	43.6	51.0	160.0	162.2
F_6-hGO	0.8	42.5	51.0	155.1	165.7
F_8-hGO	0.9	41.1	51.0	146.6	163.0
F_{10}-hGO	1.1	38.1	51.0	139.3	159.4

^{cal} Calculated value

^{exp} Experimental value

Supplementary Table 2. Comparison of antifouling performances of the F-hGO membranes and the reported GO-based membranes.

Membrane	Initial flux (L m^{-2} h^{-1})	DR_t (%)	FRR (%)	Ref.
PGS/GO	1933	38.0	93.0	[4]
PVDF/RGO@SiO_2/PDA	~132	70.0	87.2	[5]
PDA/RGO/HNTs	~70	33.0	90.9	[6]
D-HNTs/GO/EDA	218	55.0	90.0	[7]
GO/MCU-C_3N_4/PVDF	1333	95.0	84.3	[8]
PEG-rGOAMs	4890	60.0	95.0	[9]
HNTs/GO	716	37.5	86.8	[10]
GO/C_3N_4@TiO_2	2268	93.0	99.7	[11]
GO/v-COF@GO	5900	61.0	94.0	[12]
F_4-hGO	308	13.9	97.2	This work
**F_6-hGO**	**310**	**6.8**	**99.8**	**This work**
F_8-hGO	314	11.3	98.2	This work
F_{10}-hGO	305	29.4	93.7	This work

Supplementary Table 3. Comparison of antifouling performances of the F_6-hGO membranes and the reported membranes for oil-water separation.

Membrane	Initial flux ($L\ m^{-2}\ h^{-1}$)	DR_t (%)	FRR (%)	Ref.
AE2311@SMA/PVDF	362	23.0	96.6	13
EVAL-SSPBs	2600	26.6	81.0	14
PVDF/PVP/copolymer	~320	20.0	99.0	15
PDMS-PEG/PVDF	285	50.0	99.0	16
PVAc@N6/SiO_2	~1347	54.0	85.0	17
ZNG-g-PVDF	~2300	50.0	100.0	18
Cu^{2+}/Alginate/PAA-g-PVDF	1550	66.4	88.3	19
CNTs-PAN	~157	20.0	100.0	20
UiO-66-NH_2@PAA	~1165	39.0	91.0	21
SiO_2-d-PK	7533	29.0	99.0	22
PVDF/F127-F_{16}-TA	330	0	100.0	23
HPAN-PEI-PFOS	220	8.3	99.3	24
**F_6-hGO**	**310**	**6.8**	**99.8**	**This work**

Supplementary Reference:

1. Owens DK, Wendt RC. Estimation of the surface free energy of polymers. *J Appl Polym Sci* **13**, 1741-1747 (1969).

2. Yang C*, et al.* Ultrathin nanofiltration membrane assembled by polyethyleneimine-grafted graphene quantum dots. *J Membr Sci* **642**, 119944 (2022).

3. Li W*, et al.* Perfluorooctanoyl chloride engineering toward high-flux antifouling polyamide nanofilms for desalination. *J Membr Sci* **644**, 120166 (2022).

4. Zhao X, Su Y, Liu Y, Li Y, Jiang Z. Free-Standing Graphene Oxide-Palygorskite Nanohybrid Membrane for Oil/Water Separation. *ACS Appl Mater Interfaces* **8**, 8247-8256 (2016).

5. Peng Y, Yu Z, Li F, Chen Q, Yin D, Min X. A novel reduced graphene oxide-based composite membrane prepared via a facile deposition method for multifunctional applications: oil/water separation and cationic dyes removal. *Sep Purif Technol* **200**, 130-140 (2018).

6. Liu Y*, et al.* A mussel-induced method to fabricate reduced graphene oxide/halloysite nanotubes membranes for multifunctional applications in water purification and oil/water separation. *Chem EngJ* **336**, 263-277 (2018).

7. Zeng G*, et al.* Novel Halloysite Nanotubes Intercalated Graphene Oxide Based Composite Membranes for Multifunctional Applications: Oil/Water Separation and Dyes Removal. *Ind Eng Chem Res* **56**, 10472-10481 (2017).

8. Shi Y*, et al.* Evaluation of self-cleaning performance of the modified g-C3N4 and GO based PVDF membrane toward oil-in-water separation under visible-light. *Chemosphere* **230**, 40-50 (2019).

9. He M, Zhang R, Zhang K, Liu Y, Su Y, Jiang Z. Reduced graphene oxide aerogel membranes fabricated through hydrogen bond mediation for highly efficient oil/water separation. *J Mater Chem A* **7**, 11468-11477 (2019).

10. Zhu Y, Chen P, Nie W, Zhou Y. Greatly Improved Oil-in-Water Emulsion Separation Properties of Graphene Oxide Membrane upon Compositing with Halloysite Nanotubes. *Water Air Soil Poll* **229**, 94 (2018).

11. Liu Y*, et al.* 2D Heterostructure Membranes with Sunlight-Driven Self-Cleaning Ability for Highly Efficient Oil-Water Separation. *Adv Funct Mater* **28**, 1706545 (2018).

12. Liu Y*, et al.* Graphene oxide membranes with an ultra-large interlayer distance through vertically grown covalent organic framework nanosheets. *J Mater Chem A* **7**, 25458-25466 (2019).

13. Xu C*, et al.* Fabrication of hyperbranched polyether demulsifier modified PVDF membrane for demulsification and separation of oil-in-water emulsion. *J Membr Sci* **602**, 117974 (2020).

14. Ma S*, et al.* Modification of Supramolecular Membranes with 3D Hydrophilic Slide-Rings for the Improvement of Antifouling Properties and Effective Separation. *ACS Appl Mater Interfaces* **11**, 28527-28537 (2019).

15. Bhalani DV, Singh Chandel AK, Trivedi JS, Roy S, Jewrajka SK. High molecular weight poly(vinyl pyrrolidone) induces hierarchical surface morphology in poly(vinylidene fluoride) membrane and facilitates separation of oil-water emulsions. *J Membr Sci* **566**, 415-427 (2018).

16. Roy S, Bhalani DV, Jewrajka SK. Surface segregation of segmented amphiphilic copolymer of poly(dimethylsiloxane) and poly(ethylene glycol) on poly(vinylidene fluoride) blend membrane for oil–water emulsion separation. *Sep Purif Technol* **232**, 115940 (2020).

17. Islam MS, McCutcheon JR, Rahaman MS. A high flux polyvinyl acetate-coated electrospun nylon 6/SiO 2 composite microfiltration membrane for the separation of oil-in-water emulsion with improved antifouling performance. *J Membr Sci* **537**, 297-309 (2017).

18. Zhu Y*, et al.* Zwitterionic Nanohydrogel Grafted PVDF Membranes with Comprehensive Antifouling Property and Superior Cycle Stability for Oil-in-Water Emulsion Separation. *Adv Funct Mater* **28**, 1804121 (2018).

19. Gao S, Zhu Y, Wang J, Zhang F, Li J, Jin J. Layer-by-Layer Construction of Cu2+/Alginate Multilayer Modified Ultrafiltration Membrane with Bioinspired Superwetting Property for High-Efficient Crude-Oil-in-Water Emulsion Separation. *Adv Funct Mater* **28**, 1801944 (2018).

20. Tian M, Liao Y, Wang R. Engineering a superwetting thin film nanofibrous composite membrane with excellent antifouling and self-cleaning properties to separate surfactant-stabilized oil-in-water emulsions. *J Membr Sci* **596**, 117721 (2020).

21. Cao J*, et al.* Self-assembled MOF membranes with underwater superoleophobicity for oil/water separation. *J Membr Sci* **566**, 268-277 (2018).

22. Zhang L*, et al.* An ultrathinin situsilicification layer developed by an electrostatic attraction force strategy for ultrahigh-performance oil–water emulsion separation. *J Mater Chem A* **7**, 24569-24582 (2019).

23. Wang F*, et al.* In situ construction of chemically heterogeneous hydrogel surfaces toward near-zero-flux-decline membranes for oil-water separation. *J Membr Sci* **594**, 117455 (2020).

24. Yu Q*, et al.* Assembly of self-cleaning perfluoroalkyl coating on separation membrane surface. *Appl Surf Sci* **496**, 143674 (2019).
